# Effects of functional groups on the energetics of CO and CO_2_ formation during secondary oxidation of coal following pre-treatment

**DOI:** 10.1039/d5ra09282e

**Published:** 2026-02-20

**Authors:** Minyang Shen, Ziwen Dong, Keyu Wang

**Affiliations:** a School of Economics and Management, Ningbo Institute of Technology Ningbo 315211 China 1316859454@qq.com 875000528@qq.com 928715589@qq.com; b Zhejiang Institute of Tianjin University Ningbo Zhejiang 315211 China

## Abstract

To investigate how functional groups introduced in coal during pre-oxidation influence the release behavior of CO and CO_2_ during subsequent secondary oxidation, experimental characterization was conducted to identify the types and relative abundances of functional groups formed at varying pre-oxidation temperatures. Concurrently, the apparent activation energy (*E*_α_) for CO and CO_2_ evolution during secondary oxidation was determined *via* kinetic analysis. A series of multiple linear regression models were then developed to quantitatively assess the relationships between *E*_α_ values for CO/CO_2_ generation and specific functional group contents – or their combinations-in pre-oxidized coal, across distinct temperature intervals of secondary oxidation. The results indicate that, under all pre-oxidation conditions examined, the *E*_α_ for CO evolution during secondary oxidation exceeds that for CO_2_. However, this gap narrows progressively with increasing pre-oxidation termination temperature. Notably, when pre-oxidation is terminated below 140 °C, the *E*_α_ for CO formation during secondary oxidation is lower than that observed during initial (unprioritized) coal oxidation – whereas the *E*_α_ for CO_2_ formation remains consistently higher than in the initial oxidation stage. Further, within the secondary oxidation temperature range of 40–170 °C, elevated concentrations and synergistic interactions between aliphatic –CH_2_/–CH_3_ groups and carbonyl (C=O) groups significantly reduce the *E*_α_ for both CO and CO_2_ evolution. However, as the secondary oxidation temperature interval is refined into narrower subranges, the dominant functional groups-and their interaction patterns-exhibit marked variation, underscoring the context-dependent nature of functional group reactivity.

## Abstract 2

The FTIR scanning curves of the functional groups in raw coal and coal samples oxidized to different temperatures.

## Graphical abstract

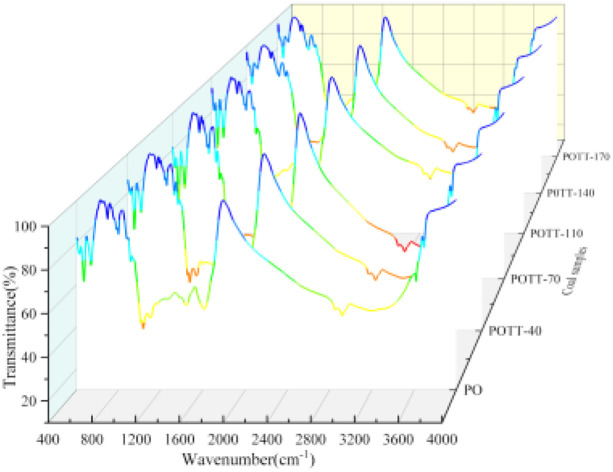

## 1 Introduction

The generation of CO_*x*_ is continuous throughout the process of coal oxidation. Therefore, the degree of coal oxidation is often characterized by CO_*x*_-related indicators. Thus, the study of CO_*x*_ plays an important role in ensuring the production safety of coal-producing and coal-consuming units and reducing carbon emissions.^1–3^ Coal spontaneous combustion (CSC) is a slow oxidation process. Most research has focused on the natural low-temperature and heating stages. CSC typically progresses until the ignition temperature is reached. However, the critical research phase for this process generally focuses on the temperature range up to 170 °C. Once this threshold is attained, in the absence of intervention, the temperature is expected to continuously increase toward the ignition point. During the low-temperature oxidation CSC process, the higher the temperature, the greater the amount of toxic and harmful gases produced. The release of CO_*x*_ is a very critical and significant gas release indicator. However, there is no precise mathematical model to describe the release law of CO_*x*_ during coal oxidation and CSC.^4–6^

Coal comprises a variety of reactive functional groups, and during the smoldering process, numerous reactions and transformations of these active groups occur. Among these gases, the generation of carbon monoxide and carbon dioxide is strongly associated with oxygen-containing functional groups.^7^ Zhao^8^ found that oxygen-containing groups are inherently the most active and reactive among all the functional groups present. Zhang^9^ proposed that the formation of CO_2_ and CO is primarily attributed to the decomposition of surface oxides formed during coal oxidation, as well as the oxygen-containing functional groups present within the coal matrix. Under low-temperature conditions, most CO_2_ emissions result from the pyrolysis of these oxygen-containing functional groups. Moreover, these functional groups significantly influence the relative production rates of CO_2_ and CO. The primary active groups involved in the coal oxidation process include bridge bonds (such as methylene groups), hydroxyl groups, and aliphatic side chains, with the hydroxyl group content having a particularly pronounced effect on the generation of both carbon dioxide and carbon monoxide.^10–12^ The activation energy (*E*_α_) associated with CO_*x*_ generation during low-temperature coal oxidation can be divided into three distinct stages based on variations in coal temperature. In general, the *E*_α_ increases with rising temperature. This variation in *E*_α_ suggests that the mechanism of CO_*x*_ formation gradually evolves as coal temperature increases, a transition closely associated with the presence and behavior of oxygen-containing functional groups.^13^ Carbonyl and carboxyl groups are key functional groups responsible for the generation of carbon monoxide and carbon dioxide. Other functional groups may also be transformed into carbonyl and carboxyl groups, which subsequently decompose into CO_*x*_. The variation in the content of these two functional groups across different coal temperature stages directly determines the amount of CO_*x*_ produced.^14^ Li^15^ conducted isothermal experiments at various temperatures combined with microscopic characterization to investigate the kinetics of CO_*x*_ generation during low-temperature oxidation. The results showed that the *E*_α_ for the formation of CO and CO_2_ under the oxidation pathway were nearly identical, suggesting that they may originate from the same precursor. It was proposed that the reaction between methyl groups in coal and O_2_ might govern the generation of carbon oxides, resulting in similar apparent activation energies for CO_*x*_ formation during the stable phase. However, studies by Aizenstat^16^ and Green^17^ have demonstrated that CO and CO_2_ originate from two parallel reaction pathways, with the activation energy for CO consistently higher than that for CO_2_. Moreover, Wang *et al.*^18,19^ conducted isothermal reaction experiments and found that, under constant temperature conditions, the activation energy for CO formation in bituminous coal is approximately 4% greater than that for CO_2_. Wang noted that during coal oxidation, both CO and CO_2_ formation require overcoming energy barriers. CO is primarily generated during the low-temperature dynamic stage, while CO_2_ is predominantly produced during the high-temperature stable stage above 70 °C. These distinct stages can be characterized by parameters such as oxygen uptake, oxygen uptake rate, activation energy, adsorption heat, heat release, mass changes, and gas evolution.^20^ The concentrations of –CH_3_/–CH_2_, –C=O, and –COOH in low-rank lignite show a significant correlation with the emissions of CO and CO_2_. For example, the reaction between C=O˙ and ˙OH radicals can occur spontaneously and generate a certain amount of heat, which may promote the CSC.^21^ According to Xu and Wang,^22,23^ the variation patterns of functional groups during the oxidation process suggest that the rapid depletion of methyl and methylene groups is primarily attributed to their reaction with O_2_, resulting in the formation of unstable intermediate products. These intermediates are further converted into thermally stable compounds, such as carbonyl and carboxyl groups, while releasing carbon dioxide gas. Using FTIR spectroscopy, Zhang^24^ investigated the relative abundance of alkyl chains and oxygen-containing functional groups during low-temperature oxidation, as well as their impact on CO_*x*_ generation. The findings revealed that, apart from water, carboxyl, carbonyl, and side-chain C–O bonds among oxygen-containing functional groups are the main contributors to CO emissions during low-temperature oxidation and serve as key active groups that facilitate CSC. The concentration of CO generated during the low-temperature oxidation of low-rank coal was strongly correlated with the relative content of –CH_3_/–CH_2_ groups, whereas CO_2_ concentration was more closely associated with the relative content of –COOH groups. Overall, the CO and CO_2_ produced during this process were primarily derived from the oxidation of alkyl chains. Specifically, alkyl chains in coal were first oxidized to carbonyl (–C=O) groups, which subsequently led to the formation of CO and carboxyl groups; these carboxyl groups then decomposed to produce CO_2_. Xin^25^ investigated the generation patterns of CO_*x*_ and their correlation with functional groups during isothermal autoignition using a combined TG-MS and FT-IR approach. The results indicated that carboxyl and carbonyl groups were the primary functional groups directly responsible for CO_*x*_ generation, while aliphatic hydrocarbons also played a crucial role as key intermediates in the formation of carboxyl and carbonyl groups. During the low-temperature oxidation stage of coal, the decomposition of oxygen-containing functional groups, together with their synergistic interaction with the oxidation of active sites, contributed to the production and release of CO_*x*_.^26^ The hydroxyl group content was found to influence the formation and relative distribution of CO_2_ and CO. However, a unified interpretation of the relationship between active group transformations and CO_*x*_ generation remains lacking.^27^

Zhang believes that if coal contains more –COOH groups and less –CH_2_ and –CH_3_, it may lead to higher thermal stability of coal.^28^ Chen points out that the decomposition of aldehyde and carboxyl groups generates more CO, and the gas desorption and high-energy oxidation of carboxyl groups cause the generation of CO_2_ to be delayed compared to CO. Temperature and functional group evolution are the key factors controlling the gas generation pathways during coal oxidation.^29^ Niu's research found that initial oxidation activates functional groups in coal, such as C–O–C, C=O, and –CH_3_, and during secondary oxidation, these functional groups are further oxidized and decomposed to accelerate the formation of gas products.^30^ Although the above studies have all clarified the qualitative relationship between functional groups and the generation of CO and CO_2_, few have studied and clarified the quantitative relationship between functional groups and the generation of CO and CO_2_, providing theoretical support for predicting and judging the generation of CO and CO_2_ under different conditions in the future. Therefore, it is necessary to conduct relevant research and detection. Based on the theory and method of multiple linear regression, the types and relative contents of functional groups under different treatments and temperatures are determined through micro-observation, and the generation amounts of CO and CO_2_ are detected macroscopically. The quantitative model between the generation amounts of CO and CO_2_ and the relative contents of multiple functional groups is calculated through regression.

Although previous studies have provided valuable insights into the smoldering combustion of coal, the gaseous products of oxidation are predominantly analyzed using gas chromatography (GC), a method associated with relatively low sensitivity and lengthy identification times for reaction products.^31^ Because of these limitations, GC is often inadequate for the analysis of trace coal samples. As a result, mass spectrometry (MS) or integrated thermogravimetric analysis coupled with mass spectrometry (TG-MS) are frequently utilized to investigate the oxidation behavior of small samples, allowing for a more precise characterization of thermal and mass changes during coal oxidation.^32–34^ Experiments involving trace samples can promote complete oxidation and enable a detailed analysis of low-temperature oxidation behavior, which holds significant value for elucidating the underlying mechanisms of low-temperature coal oxidation. However, under real-world production conditions, coal typically exhibits a broad particle size distribution. The heating process from low-temperature oxidation to ignition primarily involves the oxidation of fine particles or the surface and near-surface layers of larger particles, which generally do not undergo full oxidation. Therefore, significant discrepancies may exist between the results derived from trace sample testing and those observed in real-world production environments. To address this issue, this study aims to use mixed coal samples with particle sizes of 10 mm or smaller to perform programmed temperature experiments simulating both macroscopic pre-oxidation and secondary oxidation stages. GC will be employed to monitor the increasing concentrations of O_2_, CO, and CO_2_ throughout the oxidation process. Additionally, the functional groups of coal samples at different stages of pre-oxidation will be analyzed. The *E*_α_ of CO_*x*_ generation during various temperature intervals in secondary oxidation will also be calculated. Furthermore, multiple regression analysis will be conducted to investigate the relationship between the *E*_α_ of CO_*x*_ generation and the evolution of functional groups, aiming to clarify the variation patterns of CO_*x*_*E*_α_ during secondary coal oxidation and its correlation with functional group transformation.

## 2 Experimental materials and methods

The coal sample used in this experiment was obtained from Donggucheng Mine in Shanxi Province and had the following industrial composition: *M*_ad_ 12.17%, *A*_ad_ 14.07%, *V*_ad_ 34.32%, and FC_ad_ 39.44%. The samples were crushed to a particle size of no more than 10 mm for the programmed temperature rise experiment and FTIR analysis. In the programmed temperature rise experiment, the coal samples were placed in a pure copper tank with an inner diameter of 39 mm and an outer diameter of 42 mm, with a sample height of approximately 240 mm. Dry air was supplied at a flow rate of 100 ml min^−1^. The temperature was increased at a rate of 0.5 °C min^−1^ from ambient temperature to the designated termination temperature. The termination temperatures for pre-oxidation were set at 40, 70, 110, 140, and 170 °C, respectively. Once the coal reached the target pre-oxidation temperature, heating was stopped, and the sample was cooled using pure nitrogen. After the coal temperature decreased too and stabilized at room temperature, the nitrogen flow was terminated. The sample processing procedure and their corresponding numbers are shown in Table 1.

**Table 1:** Sample processing procedure and numbering

Whether or not it has undergone initial oxidation	Pre-oxidation temperature	Will a secondary oxidation be performed following the pre-oxidation process	Secondary oxidation termination temperature	Sample number
Yes	170	No	No	PO
Yes	40	Yes	170 °C	POTT-40
Yes	70	Yes	170 °C	POTT-70
Yes	110	Yes	170 °C	POTT-110
Yes	140	Yes	170 °C	POTT-140
Yes	170	Yes	170 °C	POTT-170

Take the coal sample and mix it thoroughly. Grind approximately 10 g of the coal sample to a particle size below 200 mesh. Dry the ground sample at 120 °C under vacuum for 2 hours and then subject it to FTIR analysis using a Nicolet iS50 Fourier Transform Infrared Spectrometer (Thermo NICOLET Company, USA). Each sample, including the original coal sample below 200 mesh, is measured twice, and the average of the two measurements is taken as the result.

The remaining coal samples were placed back into experimental vessels and subjected to secondary oxidation under a controlled heating rate of 0.5 °C min^−1^ and a dry air flow rate of 100 mL min^−1^. The secondary oxidation process was terminated at 170 °C for all samples. Gas samples were collected at 10 °C intervals, starting from 40 °C, throughout the oxidation process. The composition and concentration of the collected gases, including O_2_, CO, CO_2_, and CH_*x*_, were analyzed using a gas chromatograph (GC9100, Beijing Purui Analytical Instrument Co., Ltd). The gas chromatography column uses an 8-meter-long, 3-millimeter-diameter TDX-01 gas chromatography column. The detector includes a dual FID, TCD, and methane conversion furnace. The temperature control range of the detector is from 5 °C above room temperature to 400 °C. The temperature control accuracy of the column chamber is 0.1 °C, and the temperature control of the conversion furnace is 360 °C. The minimum detection limits for various gas components are as follows: CH_4_ < 0.5 × 10^−6^, C_2_H_4_ < 0.5 × 10^−6^, C_2_H_6_ < 0.5 × 10^−6^, C_2_H_2_ < 0.5 × 10^−6^, C_3_H_8_ < 1 × 10^−6^, CO < 1 × 10^−6^, CO_2_ < 2 × 10^−6^, O_2_ < 0.1%. Before and during the detection of oxidized gas, standard gases produced by Anhui Qiangyuan Gas Co., Ltd are generally used for calibration. The concentrations of the gas components are: CO_2_, O_2_, CO, CH_4_, C_2_H_2_, C_2_H_4_, C_2_H_6_, C_3_H_8_, N_2_, with concentrations of 407 × 10^−6^, 20.2%, 90.8 × 10^−6^, 704 × 10^−6^, 50.8 × 10^−6^, 211 × 10^−6^, 52.8 × 10^−6^, 95.6 × 10^−6^, and the remaining amounts.

## 3 Result analysis and discussion

### 3.1. The variation law of CO_*x*_ concentration

**Fig. 1:**
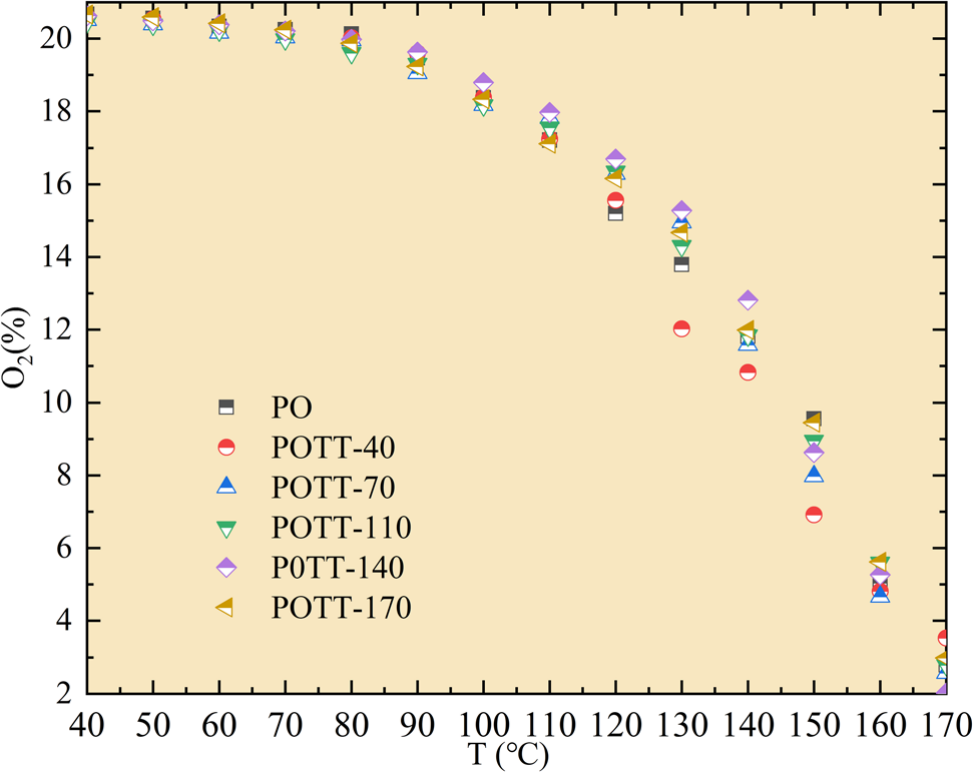
Variation pattern of O_2_ concentration with temperature during the oxidation process.

As shown in Fig. 1, during the secondary oxidation (SO) of coal samples pre-oxidized at different temperatures, the consumption of O_2_ concentration shows minimal variation within the temperature range of 40–100 °C. In the temperature range of 100–130 °C, O_2_ consumption during SO of coal samples pre-oxidized at different temperatures may increase compared to the pre-oxidation stage. However, differences in O_2_ concentration during SO still exist depending on the pre-oxidation temperature (POT). Within the experimental temperature range of 130–170 °C, the O_2_ consumption during secondary oxidation is lower than that observed during the pre-oxidation process. Furthermore, within this temperature range, the O_2_ concentration during SO decreases as the POT increases.

**Fig. 2:**
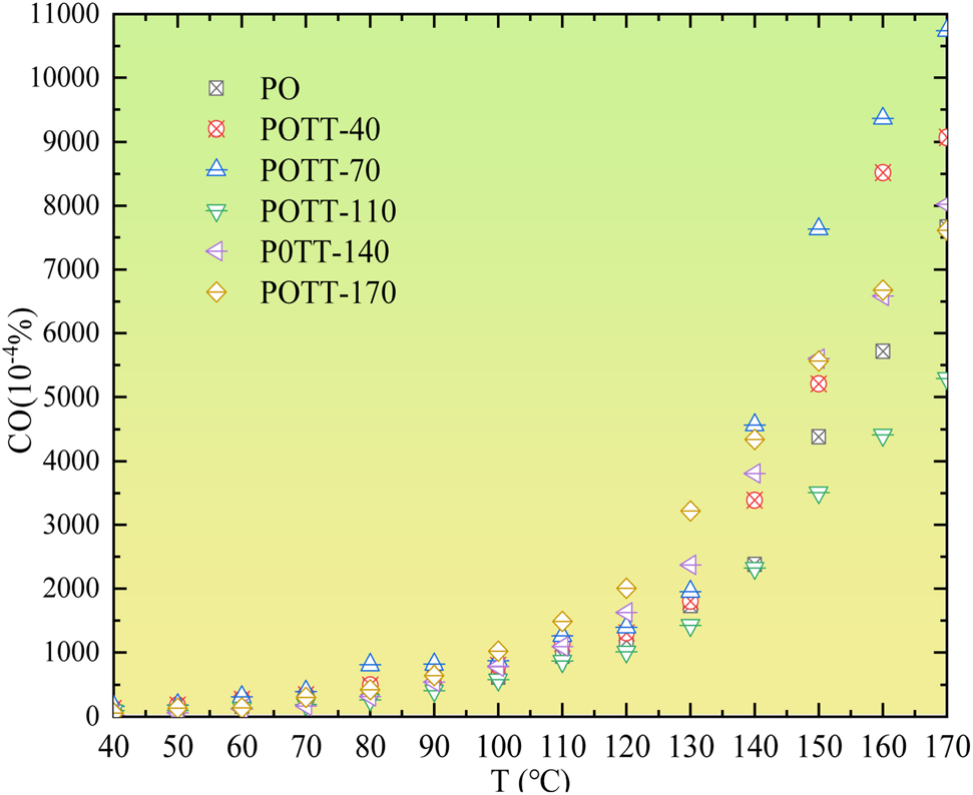
Variation pattern of CO concentration with temperature during the oxidation process.

As shown in Fig. 2, during the low temperature SO of coal samples that were pre-oxidized at different temperatures, only the sample pre-oxidized at 70 °C exhibits a slight increase in CO concentration within the temperature range of 40–90 °C. No significant changes are observed in samples pre-oxidized at other temperatures. Within the temperature range of 90–170 °C, only the sample pre-oxidized at 110 °C shows a decrease in CO concentration during SO compared to the initial oxidation stage. For all other POTs, the CO concentration during SO is higher than that observed during the initial oxidation process.

**Fig. 3:**
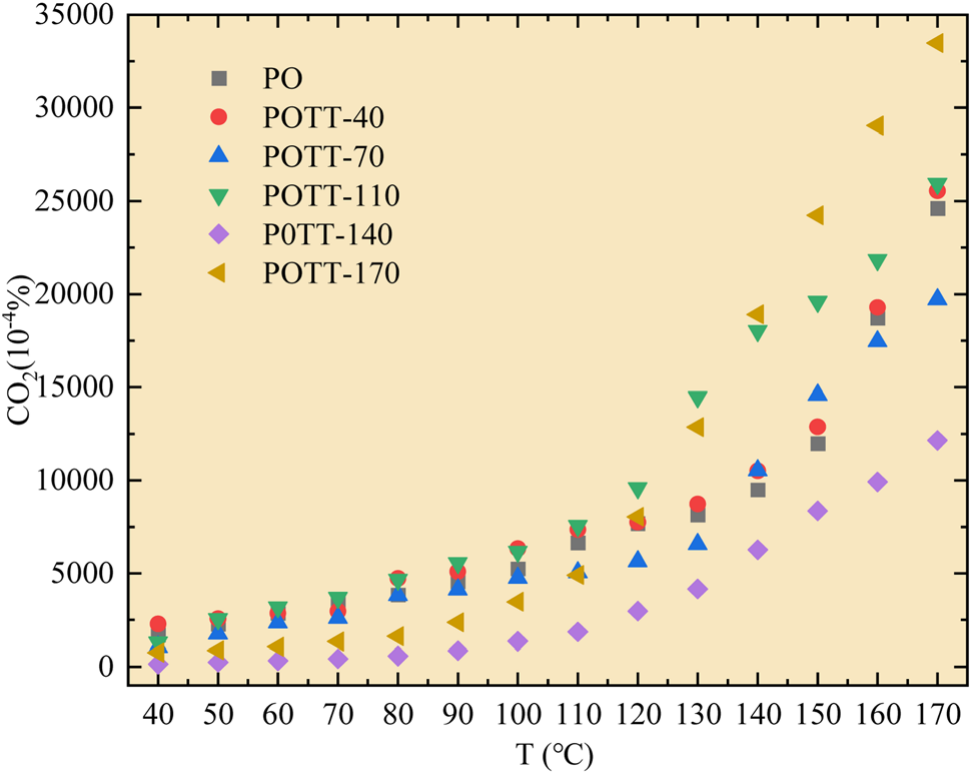
Concentration of CO_2_ during the oxidation process varies with temperature.

As shown in Fig. 3, during the low-temperature SO of coal samples pre-oxidized at different temperatures, the CO_2_ concentration within the temperature range of 40–110 °C was significantly lower compared to the pre-oxidation stage when the POT is 140 °C or 170 °C. However, for samples pre-oxidized at 40 °C, 70 °C, and 110 °C, no notable differences in CO_2_ concentration were observed compared to the pre-oxidation process. When the temperature exceeded 110 °C, the variation in CO_2_ concentration during SO became more pronounced relative to the pre-oxidation stage. Notably, in the case of coal samples pre-oxidized to 170 °C, a significant increase in CO_2_ concentration was observed during SO once the temperature surpassed 110 °C.

### 3.2. The variation law of CO_*x*_ generation rate

Based on the variation trends of O_2_, CO, and CO_2_ concentrations with temperature, as illustrated in Fig. 1–3, and combined with eqn (1)–(3), the O_2_ consumption rate, CO generation rate, and CO_2_ generation rate of the coal sample can be calculated. By taking the logarithm of both sides of the Arrhenius equation (eqn (4)) and (5) can be derived. According to eqn (5), the *E*_α_ for CO_*x*_ generation can be determined through linear regression analysis.1
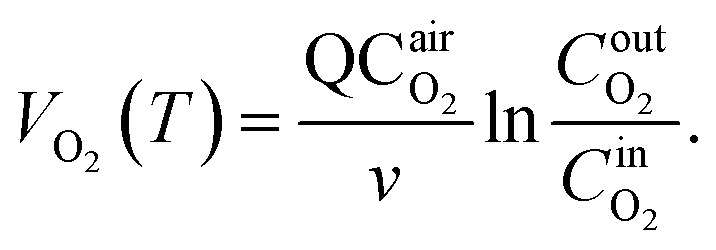
2
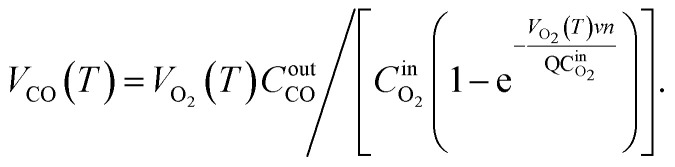
3
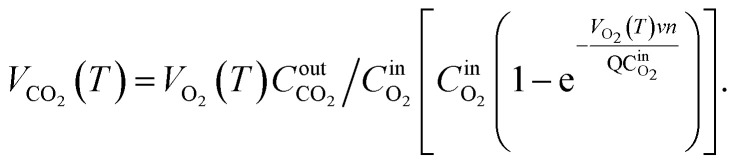
4
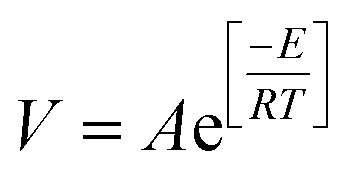
5
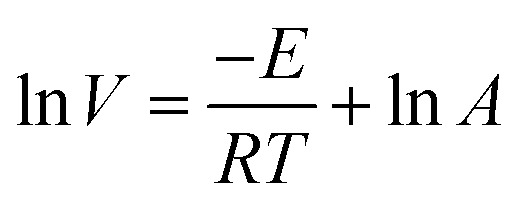


Note: in eqn (1)–(11), *V*_O_2__(*T*)is the O_2_ consumption rate of the coal in the air environment or for an O_2_ concentration of 21% (mol mL^−1^ s^−1^); and *C*_O_2__^air^is the O_2_ concentration in the fresh air environment or for an O_2_ concentration of 21%. The *C*_O_2__^out^ and *C*_O_2__^in^ are the O_2_ concentrations at the outflow and inflow, respectively (%), the O_2_ concentration of the intake air is 20.96%. *v* is the volume of the coal sample (cm^3^).*Q* is the rate inflow of the airflow (mL min^−1^).

**Fig. 4:**
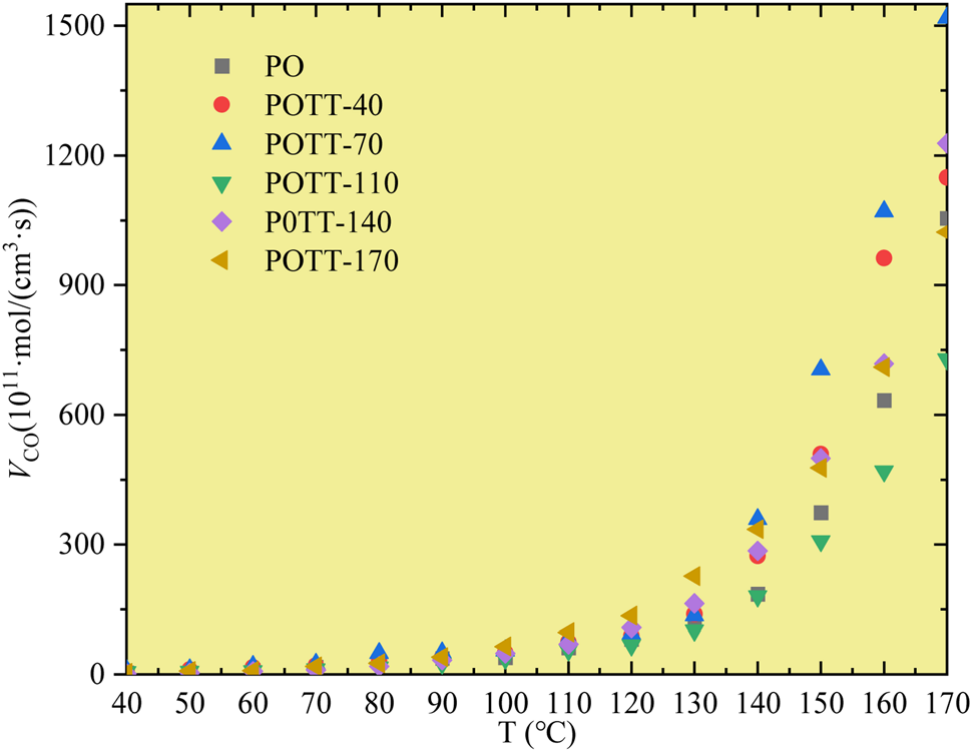
Variation of CO generation rate with temperature.

The CO generation rate is shown in Fig. 4. As can be observed, when the coal temperature is below 110 °C, the CO generation rates of different coal samples remain relatively consistent, irrespective of whether they undergo primary or SO. However, once the coal temperature surpasses 110 °C, the differences in CO generation rates among the samples become progressively more significant. Overall, the sample that underwent SO after being pre-oxidized to 70 °C exhibits the highest CO generation rate. The rates subsequently decreased in the following order, samples pre-oxidized to 40 °C, 140 °C, and 170 °C. These values are consistently higher than those observed during primary oxidation. Notably, only the sample subjected to SO after pre-oxidation to 110 °C displays the lowest CO generation rate, which is even lower than that of the primary oxidation process.

**Fig. 5:**
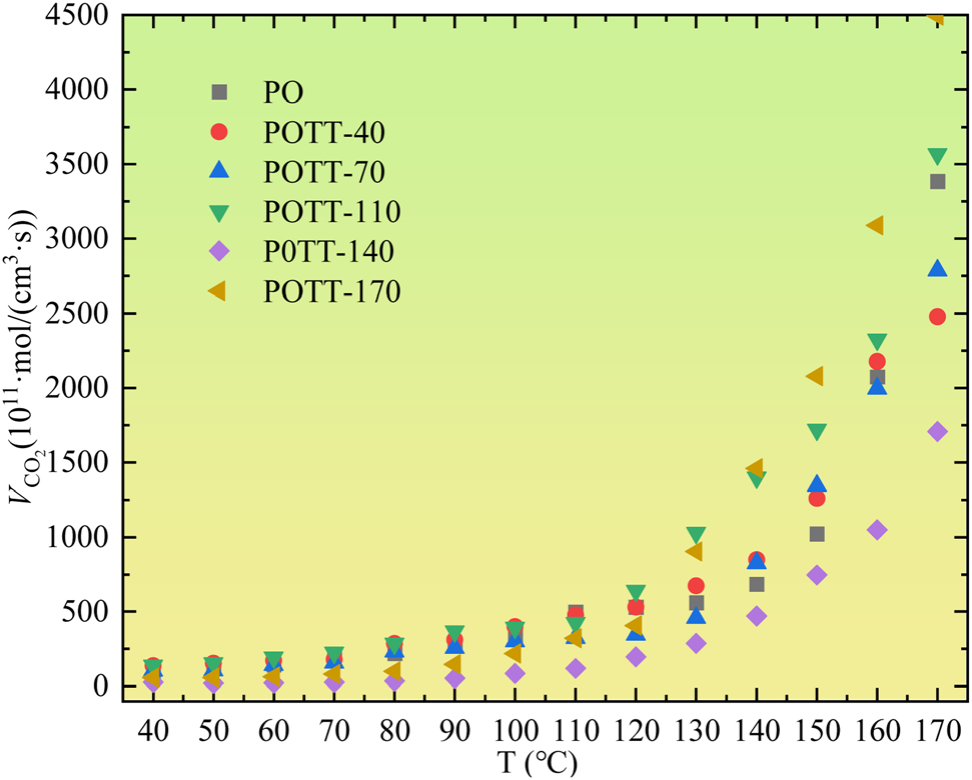
Variation of CO_2_ generation rate with temperature.

The variation in CO_2_ concentration with temperature differs slightly from that of CO. While a detectable amount of CO appears at the onset of oxidation, the abrupt increase in CO_2_ generation rate becomes evident only after the coal temperature exceeds 110 °C, as shown in Fig. 5. During the oxidation stage characterized by a relatively high CO_2_ generation rate, the coal samples subjected to SO following initial oxidation to termination temperatures of 170 °C and 110 °C exhibit the highest CO_2_ generation rates. The next highest rates are observed in samples undergoing primary oxidation only. Subsequently, lower CO_2_ generation rates are found in samples that undergo SO after initial oxidation (IO) to 70 °C and 40 °C. The lowest CO_2_ generation rate is observed in the sample subjected to SO after initial oxidation to 140 °C.

### 3.3. Variation law of activation energy for CO_*x*_ generation

#### 3.3.1. Variation law of activation energy for CO generation

**Fig. 6:**
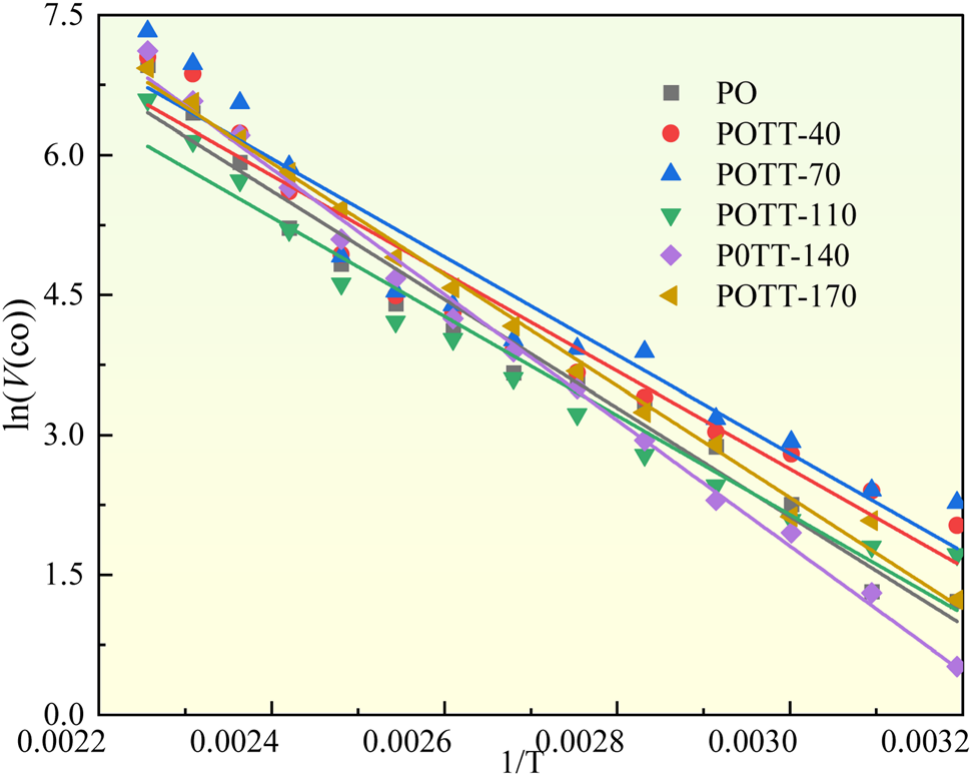
Linear fitting of the activation energy for CO generation in the coal temperature range of 40–170 °C.

The *E*_α_ fitting process for CO generation throughout the entire experimental stage, conducted at coal temperatures ranging from 40 to 170 °C, is presented in Fig. 6, with the corresponding fitting results summarized in Table 2. The *E*_α_ for CO generation varies across coal samples subjected to different termination temperatures of the IO. Compared to the IO phase, the *E*_α_ of CO generation during SO decreases by 9–10% in coal samples with IO termination temperatures of 40, 70, and 110 °C. In contrast, for samples terminated at 140 and 170 °C, the *E*_α_ increases by 16% and 3%, respectively. Specifically, when the IO temperature is below 110 °C, the *E*_α_ for CO generation during SO decreases significantly. However, when the IO temperature exceeds 140 °C, the *E*_α_ increases during SO.

**Table 2:** Activation energy of CO generation in different coal samples at 40–170 °C

Temperature	Coal sample	Intercept	Slope	*R* ^2^	*E*	Rate of change to PO
40–170 °C	PO	19.59	−5820.37	0.9703	48.39	0.0%
	POTT-40	18.37	−5243.60	0.9421	43.60	−10%
	POTT-70	18.61	−5269.71	0.9243	43.81	−9%
	POTT-110	18.08	−5313.13	0.9610	44.17	−9%
	P0TT-140	22.04	−6744.70	0.9940	56.08	16%
	POTT-170	20.27	−5980.04	0.9938	49.72	3%

As outlined above, the entire experimental testing phase encompassing secondary coal oxidation over a temperature range of 40–170 °C was partitioned into four characteristic temperature intervals, as summarized in Table 3, to support a more rigorous and systematic comparative analysis.

**Table 3:** Division and numbering of different coal temperature stages of CO_*x*_

The stage of secondary oxidation of coal temperature (°C)	40–170	40–70	70–110	110–140	140–170
Numbers of activation energies for CO activation in different temperature ranges	S_OP_-CO	S_1_-CO	S_2_-CO	S_3_-CO	S_4_-CO
Numbers of activation energies for CO_2_ activation in different temperature ranges	S_OP_-CO_2_	S_1_-CO_2_	S_2_-CO_2_	S_3_-CO_2_	S_4_-CO_2_

**Fig. 7:**
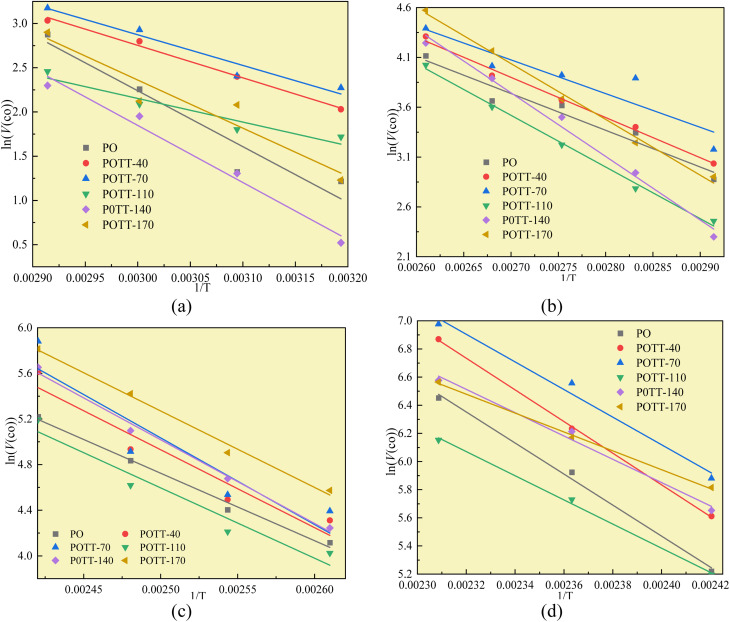
The fitting process of CO generation activation energy at different temperature stages (a) coal temperature stage of 40–70 °C, (b) coal temperature stage of 70–110 °C, (c) coal temperature stage of 110–140 °C, (d) coal temperature stage of 140–170 °C.

Fig. 7 illustrates the fitting process of *E*_α_ for CO generation at various temperature stages during the SO of coal. Based on these fitting results, the *E*_α_ for CO generation during the SO of different coal samples within the temperature ranges of 40–70 °C, 70–110 °C, 110–140 °C, and 140–170 °C were calculated and are summarized in Tables 2 and 4–6, respectively.

The *E*_α_ for the generation of CO during the IO at 40–70 °C is 52.54 kJ mol^−1^, as shown in Table 4. When the coal samples that have undergone IO at 40, 70, and 110 °C are subjected to SO, the *E*_α_ for CO generation in the 40–70 °C stage decreases by 42%, 45%, and 58% respectively indicates that when the IO is terminated at a temperature below 110 °C, the coal samples undergo SO, and the *E*_α_ for CO generation in the 40–70 °C stage is significantly lower than that of the IO under the same conditions. Moreover, as the termination temperature of the IO increases, the reduction in the *E*_α_ also increases. This suggests that the energy required to produce CO is lower after the initial oxidation. However, when the IO is terminated at 140 °C and SO occurs, the *E*_α_ for CO generation in the 40–70 °C stage is only slightly higher than that of the IO by 2%. When the IO is terminated at 170 °C and SO occurs, the *E*_α_ for CO generation in the 40–70 °C stage is 14% lower than that of the IO.

**Table 4:** Activation energy of CO generation in the 40–70 °C stage of secondary oxidation

Temperature	Coal sample	Intercept	Slope	*R* ^2^	*E*	Rate of change to PO
40–70 °C	PO	21.20	−6318.92	0.8786	52.54	0%
	POTT-40	13.76	−3669.20	0.9898	30.51	−42%
	POTT-70	13.26	−3461.25	0.9230	28.78	−45%
	POTT-110	10.17	−2671.10	0.8846	22.21	−58%
	P0TT-140	21.20	−6452.07	0.9720	53.64	2%
	POTT-170	18.68	−5438.84	0.8728	45.22	−14%

As shown in Table 5, during the coal temperature range of 70–110 °C, except for the coal sample with the IO termination temperature of 70 °C, which has an 8% reduction in the *E*_α_ for the generation of CO during the SO, the *E*_α_ for the formation of CO during the SO of the other coal samples with different IO termination temperatures all significantly increase.

**Table 5:** Activation energy of CO generation in the 70–110 °C stage of secondary oxidation

Temperature	Coal sample	Intercept	Slope	*R* ^2^	*E*	Rate of change to PO
70–110 °C	PO	13.69	−3686.75	0.9294	30.65	0%
	POTT-40	14.79	−4031.55	0.9892	33.52	9%
	POTT-70	13.21	−3382.25	0.8004	28.12	−8%
	POTT-110	17.52	−5186.89	0.9938	43.12	41%
	P0TT-140	20.98	−6383.33	0.9871	53.07	73%
	POTT-170	19.16	−5600.23	0.9916	46.56	52%

As shown in Table 6, during the coal temperature range of 110–140 °C, the *E*_α_ for the generation of CO through SO of all coal samples significantly increases. This indicates that the energy required for the SO of coal to produce CO at this temperature stage is significantly higher than that at the same temperature stage during the primary oxidation.

**Table 6:** Activation energy of CO generation in the 110–140 °C stage of secondary oxidation

Temperature	Coal sample	Intercept	Slope	*R* ^2^	*E*	Rate of change to PO
110–140 °C	PO	19.51	−5916.07	0.9865	49.19	0%
	POTT-40	22.01	−6832.59	0.8913	56.81	15%
	POTT-70	24.03	−7597.78	0.7814	63.17	28%
	POTT-110	20.02	−6167.68	0.9149	51.28	4%
	P0TT-140	23.37	−7339.66	0.9887	61.02	24%
	POTT-170	22.03	−6703.56	0.9873	55.73	13%

As shown in Table 7, during the coal temperature stage of 140–170 °C, the *E*_α_ for the generation of CO during the SO of all coal samples decreases significantly except for the coal sample that undergo SO after pre-oxidation to 40 °C. Moreover, the proportion of reduction increases significantly as the termination POT increases.

**Table 7:** Activation energy of CO generation in the 140–170 °C stage of secondary oxidation of coal samples

Temperature	Coal sample	Intercept	Slope	*R* ^2^	*E*	Rate of change to PO
140–170 °C	PO	31.94	−11030.47	0.9905	91.71	0%
	POTT-40	32.84	−11250.57	0.9992	93.54	2%
	POTT-70	29.67	−9813.45	0.9710	81.59	−11%
	POTT-110	25.99	−8584.38	0.9940	71.37	−22%
	P0TT-140	25.73	−8281.20	0.9762	68.85	−25%
	POTT-170	22.04	−6708.03	0.9957	55.77	−39%

#### 3.3.2. Variation law of activation energy for CO_2_ generation

**Fig. 8:**
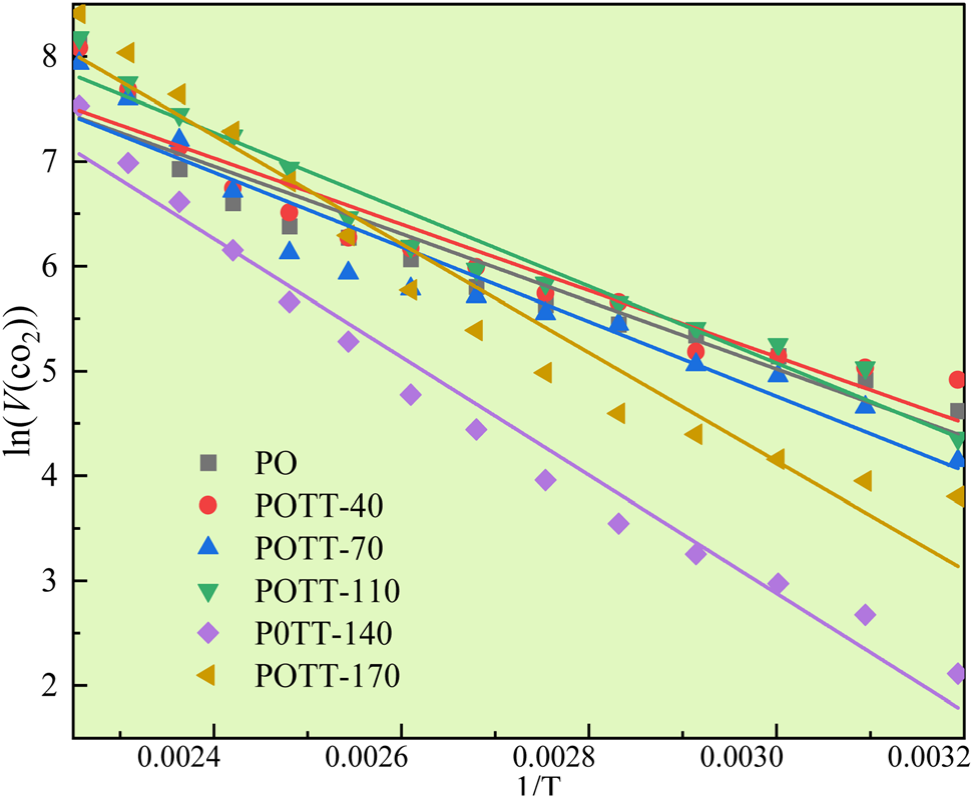
The fitting process of activation energy for CO_2_ generation in the temperature range of 40–170 °C.

The fitting process and results of the *E*_α_ of CO_2_ production in different coal samples during the 40–170 °C stage are shown in Fig. 8 and Table 8. The results indicate that, compared with the IO, except for the coal sample that underwent SO at 40 °C after the IO, where the *E*_α_ of CO_2_ production decreased by 2%, the *E*_α_ of CO_2_ production during the SO after the IO at 70 °C, 110 °C, 140 °C, and 170 °C increased by 11%, 14%, 75%, and 61% respectively. This suggests that the energy consumed for CO_2_ production during the SO of coal samples after different degrees of IO will significantly increase.

**Table 8:** The activation energy for CO_2_ generation in the coal temperature range of 40–170 °C stage

Temperature	Coal sample	Intercept	Slope	*R* ^2^	*E*	Rate of change to PO
0–170 °C	PO	14.69002	−3223.3	0.90553	26.80	0%
	POTT-40	14.61403	−3159.19	0.91142	26.27	−2%
	POTT-70	15.44661	−3562.62	0.92006	29.62	11%
	POTT-110	16.06605	−3663.17	0.96159	30.46	14%
	P0TT-140	19.79523	−5638.56	0.97476	46.88	75%
	POTT-170	19.6792	−5179.74	0.94659	43.06	61%

**Fig. 9:**
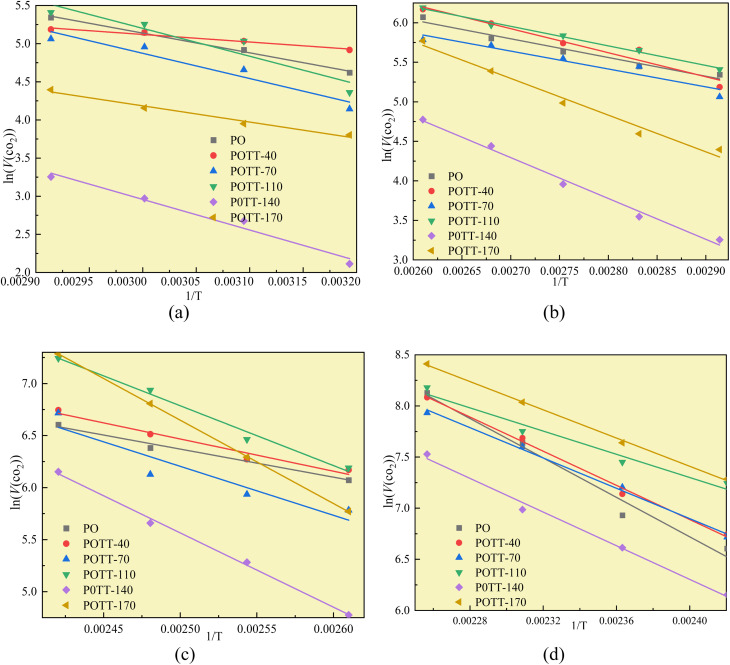
The fitting process of activation energy for CO_2_ generation at different temperature stages (a) coal temperature stage of 40–70 °C, (b) coal temperature stage of 70–110 °C, (c) coal temperature stage of 110–140 °C, (d) coal temperature stage of 140–170 °C.

The fitting process of the *E*_α_ for the generation of CO_2_ in coal samples during the IO and SO at different degrees of IO at the temperature stages of 40–70, 70–110, 110–140, and 140–170 °C is shown in Fig. 9(a–d), and the results of the *E*_α_ solution are presented in Tables 9–12.

As shown in Table 9, the *E*_α_ of CO_2_ generated during the 40–70 °C stage of SO after the IO to 40, 70, 110, 140, and 170 °C is compared with the temperature stage of the IO. The *E*_α_ of CO_2_ generated during this temperature stage of the coal sample after the IO to 40 and 170 °C is reduced by 62% and 18% respectively compared to the IO stage. The *E*_α_ of CO_2_ generated during the IO to 70, 110, and 140 °C is increased by 28%, 41%, and 55% respectively compared to the IO stage. As shown in Table 10, the *E*_α_ of CO_2_ generated by coal samples undergoing SO after IO to different temperatures during the 70–110 °C stage decreased by 5% compared to the IO stage at 70 °C. However, other coal samples with different IO termination temperatures showed varying degrees of decrease compared to the IO stage. As shown in Table 11, the *E*_α_ of CO_2_ generated by coal samples undergoing SO during the coal temperature range of 110–140 °C is significantly increased compared to the IO stage. As shown in Table 12, during the coal temperature stage of 140–170 °C, the *E*_α_ of CO_2_ generated by SO decreased by 13%, 23%, 41%, 15%, and 28% respectively compared to the same temperature stage of primary oxidation. This indicates that in the process of coal undergoing different degrees of primary oxidation and then undergoing SO, when the coal temperature rises to a higher temperature stage of 140–170 °C, the *E*_α_ of CO_2_ generated will be significantly lower than that of IO, and the proportion of *E*_α_ reduction in this temperature stage where SO occurs after IO to 110 °C is the largest.

**Table 9:** The activation energy for CO_2_ generation in the coal temperature of 40–70 °C stage

Temperature	Coal sample	Intercept	Slope	*R* ^2^	*E*	Rate of change to PO
40–70 °C	PO	12.88487	−2582.24	0.99348	21.47	0%
	POTT-40	8.08965	−989.713	0.95854	8.23	−62%
	POTT-70	14.79108	−3305.89	0.89879	27.49	28%
	POTT-110	16.1617	−3653.71	0.84951	30.38	41%
	P0TT-140	14.99959	−4013.89	0.96478	33.37	55%
	POTT-170	10.56224	−2125.8	0.97456	17.67	−18%

**Table 10:** The activation energy for CO_2_ generation in the coal temperature of 70–110 °C stage

Temperature	Coal sample	Intercept	Slope	*R* ^2^	*E*	Rate of change to PO
70–110 °C	PO	12.19121	−2368.14	0.95618	19.69	0%
	POTT-40	14.13502	−3040.68	0.93685	25.28	28%
	POTT-70	11.74744	−2261.51	0.89532	18.80	−5%
	POTT-110	12.63168	−2472.87	0.99112	20.56	4%
	P0TT-140	18.24551	−5167.16	0.98749	42.96	118%
	POTT-170	17.83019	−4642.29	0.97498	38.60	96%

**Table 11:** The activation energy for CO_2_ generation during the coal temperature of 110–140 °C stage

Temperature	Coal sample	Intercept	Slope	*R* ^2^	*E*	Rate of change to PO
110–140 °C	PO	13.11572	−2698.24	0.97298	22.43	0%
	POTT-40	14.20797	−3096.31	0.94946	25.74	15%
	POTT-70	17.96904	−4705.84	0.81814	39.12	74%
	POTT-110	21.15388	−5746.96	0.98178	47.78	113%
	P0TT-140	23.40879	−7137.73	0.99532	59.34	165%
	POTT-170	26.6729	−8009.98	0.99992	66.60	197%

**Table 12:** The activation energy for CO_2_ generation in the coal temperature range of 140–170 °C stage

Temperature	Coal sample	Intercept	Slope	*R* ^2^	*E*	Rate of change to PO
140–170 °C	PO	29.85032	−9637.54	0.96891	80.13	0%
	POTT-40	26.93024	−8350.48	0.99268	69.43	−13%
	POTT-70	24.68235	−7410.11	0.99379	61.61	−23%
	POTT-110	20.91729	−5673.86	0.95258	47.17	−41%
	P0TT-140	26.05457	−8229.45	0.98956	68.42	−15%
	POTT-170	23.9705	−6900.32	0.99785	57.37	−28%

In the complete experimental stage of 40–170 °C, except for a slight decrease of 2% in the overall *E*_α_ of CO_2_ generated during the SO process of coal samples with an IO termination temperature of 40 °C, the overall *E*_α_ of CO_2_ generated by coal samples with different oxidation termination temperatures significantly increased. After dividing the complete experimental stage of 40–170 °C into four different reaction stages, only when the coal temperature increased to 140–170 °C, the *E*_α_ of CO_2_ generated by the SO temperature significantly decreased to varying degrees (decreased by 13–41%), and other temperature stages significantly increased.

**Fig. 10:**
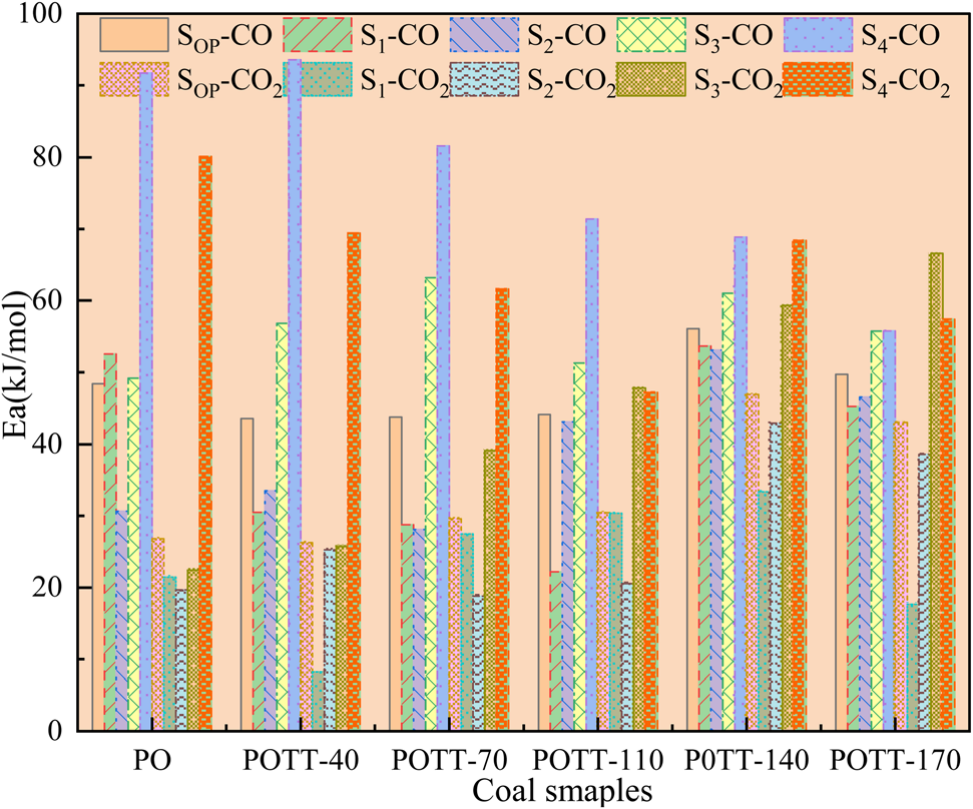
The activation energy of CO and CO_2_ produced by coal at different temperature stages. Note: S_OP_-CO, S_1_-CO, S_2_-CO, S_3_-CO, S_4_-CO respectively represents the average activation energy for the generation of CO during the complete stage of secondary oxidation at coal temperatures 40–170, 40–70 °C, 70–110, 110–140, 140–170 °C. S_OP_-CO_2_, S_1_-CO_2_, S_2_-CO_2_, S_3_-CO_2_, S_4_-CO_2_ these numbers represent the activation energy of CO_2_, and the temperature stages correspond to those of the previous CO.

Fig. 10 shows the *E*_α_ for the generation of CO and CO_2_ from different coal samples at different temperature stages. CO requires higher energy to break the C–O bond or reorganize oxygen-containing functional groups, while CO_2_ may come from more easily dissociated carboxyl or carbonyl groups, resulting in the corresponding *E*_α_ for CO generation always being higher than that for CO_2_ generation under the same coal sample conditions (except for a few cases). During the experimental process in this paper, the *E*_α_ for CO generation at the coal temperature stage of 40–170 °C was significantly higher than that for CO_2_ generation. The *E*_α_ for CO_2_ generation during the pre-oxidation stage was 55% of the *E*_α_ for CO generation. After SO of the coal samples with POT of 40, 70, 110, 140, and 170 °C, the *E*_α_ for CO_2_ generation was 60%, 68%, 69%, 84%, and 87% of the *E*_α_ for CO generation, respectively. That is, as the termination temperature of the IO increases, the difference in *E*_α_ for the generation of CO and CO_2_ during SO gradually decreases.

In conclusion, when coal samples pre-oxidized to the same termination temperature undergo SO under identical temperature conditions, the *E*_α_ for CO generation is consistently higher than that for CO_2_. However, this difference decreases as the POT increases. In the coal temperature range of 40–170 °C, 140 °C serves as a critical threshold. If the POT is below 140 °C, the *E*_α_ for CO during SO is about 10% lower than during IO. In contrast, for samples pre-oxidized above 140 °C, the *E*_α_ for CO_2_ during SO is generally 11–75% higher than during IO. Among all samples, the one pre-oxidized at 140 °C shows the highest *E*_α_ for CO_2_ during SO. During the SO of pre-oxidized coal samples, *E*_α_ for CO and CO_2_ generation is generally higher than during pre-oxidation, except in specific temperature ranges. Specifically, when pre-oxidation ends at 40 °C and 170 °C, the *E*_α_ for CO_2_ generation decreases by 62% and 18%, respectively, compared to the corresponding pre-oxidation stages. In the 40–70 °C, 70–110 °C, and 110–140 °C ranges, *E*_α_ remains higher during SO. However, in the 140–170 °C range, *E*_α_ for both CO and CO_2_ is lower during SO than during pre-oxidation.

#### 3.3.3. Correlation between the activation energy of CO_*x*_ generation and functional groups

**Fig. 11:**
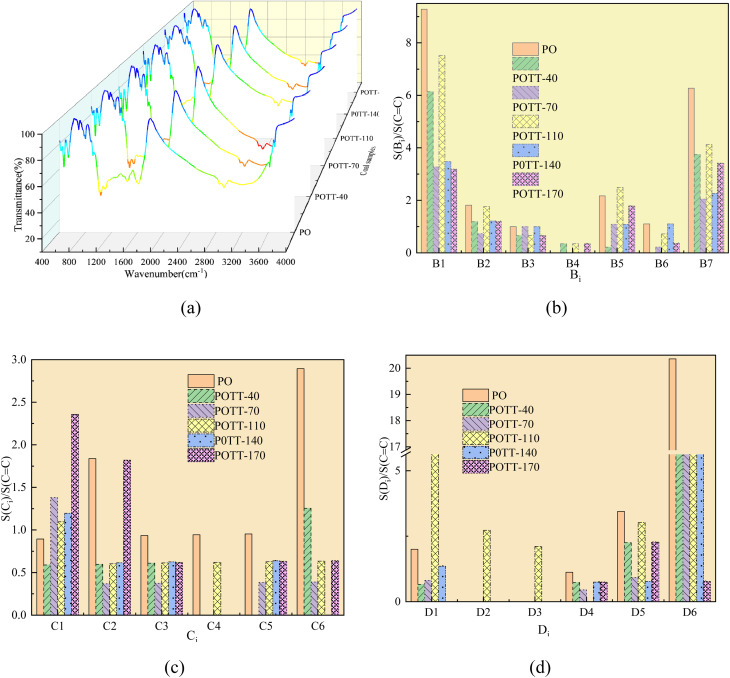
Infrared spectra of coal samples and relative contents of functional groups (a) FTIR scanning curve, (b) relative content of oxygen-containing functional groups, (c) relative content of aliphatic functional groups, (d) relative content of hydroxyl functional groups.

The FTIR scanning curves of the functional groups in raw coal and coal samples oxidized to different temperatures are shown in Fig. 11(a). The infrared spectra of the coal samples were analyzed by PeakFit V4.2 software to obtain the different classifications of functional groups (the classification basis of functional groups is shown in Table 13) and their area data in the coal samples.^35–37^ Considering that the C=C structure in coal basically does not change during the low-temperature oxidation process, the relative contents of different functional groups in the coal samples were obtained by dividing the area of other functional groups detected in each sample by the area of C=C, as shown in Fig. 11(b–d).

**Table 13:** Functional group classification in FTIR detection data

Classification	Wave number	Functional group	Serial number
Oxygen-containing functional groups	1030–1330	Ar–C–O–	*B*1
	1365–1465	–CH_2_, –CH_3_	*B*2
	1465–1590	C=C	*B*3
	1595–1635	C=C	*B*4
	1640–1690	quin. C=O	*B*5
	1690–1700	car. C=O	*B*6
	1705–1800	*R*_2_ C=O, *ν* C = O, R_COOH_, Ar_COOH_, R_COO_ Ar, *etc.* C=O	*B*7
Aliphatic functional groups	2350–2780	–COOH	*C*1
	2830–2855	*sym*. –CH_2_	*C*2
	2862–2882	*sym*. –CH_3_	*C*3
	2900	–CH	*C*4
	2918–2935	*asym*. –CH_2_	*C*5
	2950–2975	*asym*. –CH_3_	*C*6
Hydroxyl functional groups	3050–3150	aro. C–H	*D*1
	3200	rh. C–H	*D*2
	3300–3315	OH⋯OR	*D*3
	3400–3440	OH⋯OH	*D*4
	3530–3545	OH⋯π	*D*5
	3610–3800	fr. –OH	*D*6

Prior to constructing the multi-dimensional numerical model, the relevant data were normalized to the interval [−1, 1] using eqn (6). This normalization serves three principal purposes: (i) eliminating scale disparities arising from heterogeneous units across indicators-such as relative abundances of functional groups and activation energies-thereby enabling equitable comparison and integration; (ii) mitigating order-of-magnitude differences that could otherwise bias model outcomes toward numerically dominant variables; and (iii) enhancing both the numerical stability and convergence rate of the subsequent computational procedure. The results are presented in Table 14. (Notably, *B*3 and *B*4 correspond to C=C bonds and were therefore excluded from the relative content analysis. Similarly, *D*2 and *D*3, which represent aro. C–H and rh. C–H bonds respectively, were only detected in the POTT-110 sample and not in others. Therefore, they should not be included in the normalization process and stepwise regression either).6
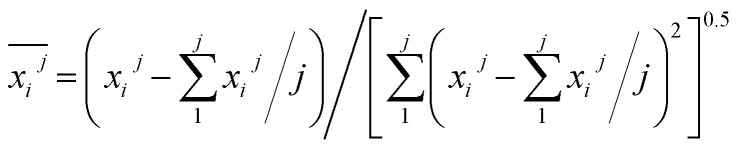

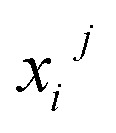
 denotes the *j*-th observed value of the *i*-th indicator.7
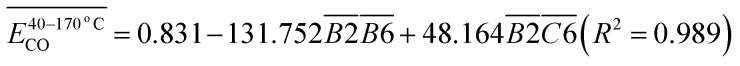


**Table 14:** The normalized processing results of the relative content of functional groups and activation energy

Planned variables	POTT-40	POTT-70	POTT-110	P0TT-140	POTT-170
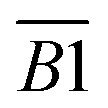	0.31	0.25	0.32	0.14	0.45
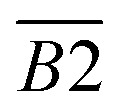	−0.07	−0.08	−0.09	−0.05	−0.03
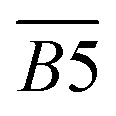	−0.14	−0.04	−0.04	−0.07	0.11
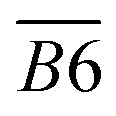	−0.16	−0.15	−0.16	−0.06	−0.23
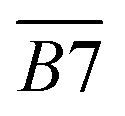	0.13	0.09	0.08	0.03	0.51
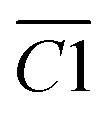	−0.11	0	−0.14	−0.06	0.25
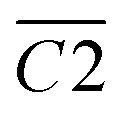	−0.11	−0.13	−0.17	−0.11	0.12
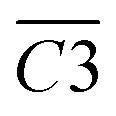	−0.11	−0.13	−0.17	−0.1	−0.17
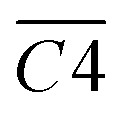	−0.16	−0.18	−0.17	−0.16	−0.32
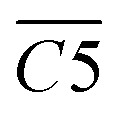	−0.16	−0.13	−0.17	−0.1	−0.17
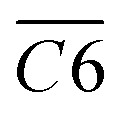	−0.06	−0.13	−0.17	−0.16	−0.17
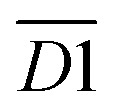	−0.11	−0.07	0.4	−0.04	−0.32
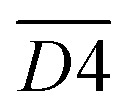	−0.1	−0.12	−0.22	−0.09	−0.14
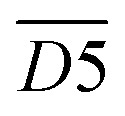	0.01	−0.06	0	−0.09	0.23
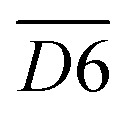	0.85	0.88	0.69	0.93	−0.13
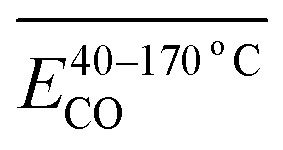	−0.37	−0.35	−0.32	0.77	0.19
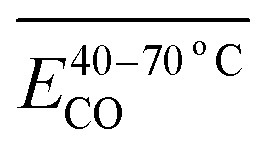	−0.28	−0.34	−0.56	0.5	0.21
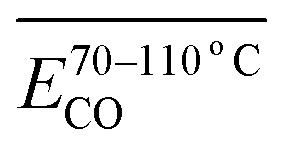	−0.26	−0.5	0.18	0.63	0.33
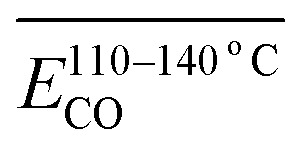	0.05	0.58	−0.41	0.4	−0.04
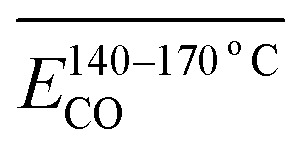	0.5	0.14	−0.18	−0.25	−0.66
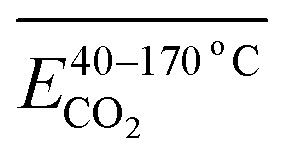	−0.38	−0.21	−0.17	0.66	0.47
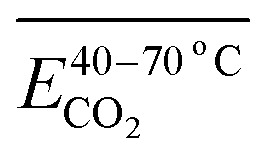	−0.72	0.21	0.35	0.49	−0.26
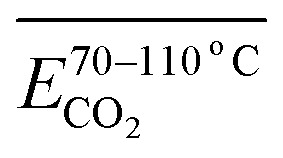	−0.1	−0.38	−0.3	0.65	0.47
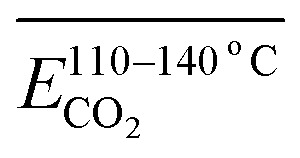	−0.45	−0.11	0.11	0.4	0.58
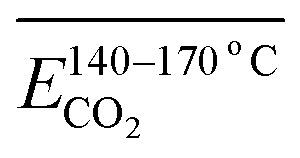	0.21	−0.1	−0.67	0.17	−0.26

During the complete low-temperature SO stage of coal at 40–170 °C in the coal SO process, the average *E*_α_ of CO generation 
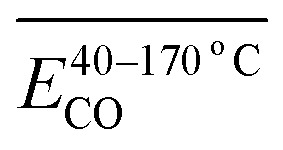
 has a relationship with 
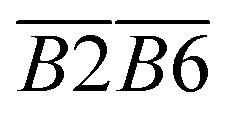
 (–CH_2_/–CH_3_ & car. C=O) and 
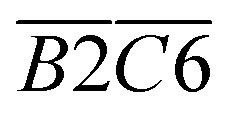
 (–CH_2_/–CH_3_ & *asym*. –CH_3_) as shown in eqn (7). The product of the relative contents of –CH_2_/–CH_3_ and car. C=O before SO is negatively correlated with the *E*_α_ and has the effect of reducing the *E*_α_. The main reason is that the –CH_2_ adjacent to the carbonyl group is prone to oxidation, and the energy barrier is reduced by forming a six-membered ring transition state. The product of the relative contents of –CH_2_/–CH_3_ and *asym*. –CH_3_ is positively correlated with the *E*_α_ for CO formation at this temperature stage, which has the effect of increasing the *E*_α_. The main reason is that the steric hindrance effect makes the oxidation of the asymmetric –CH_3_ require higher energy.^38–41^ The main process of eqn (7) is shown in Fig. 12.8
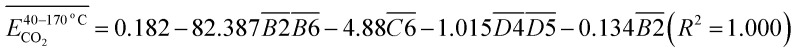


**Fig. 12:**
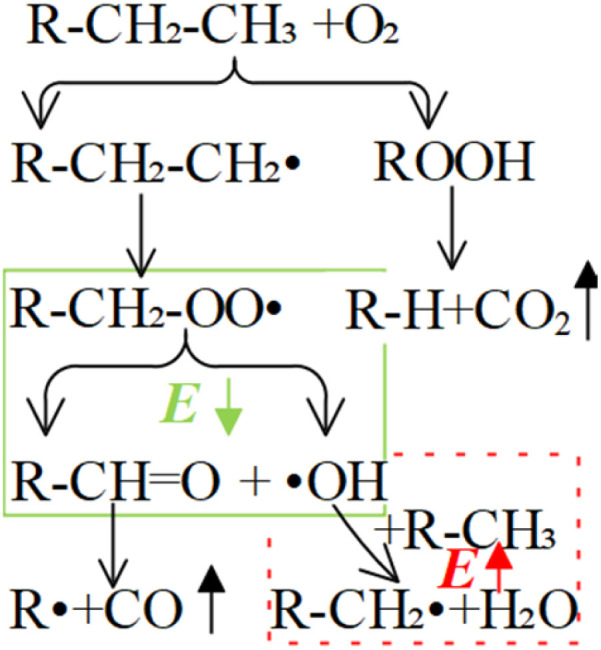
The main reaction pathway of eqn (7).

During the complete low-temperature SO stage of coal at 40–170 °C in the coal SO process, the average *E*_α_ of CO_2_ generation 
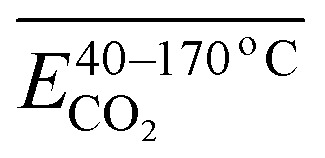
 is related to 
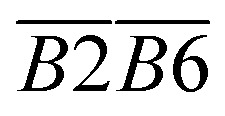
 (–CH_2_/–CH_3_ & car. C=O), 
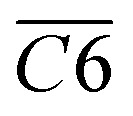
 (*asym*. –CH_3_) 
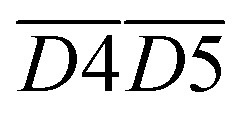
 (OH⋯OH & OH⋯π) and 
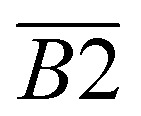
 (–CH_2_/–CH_3_) as shown in eqn (8). The relative content of the functional groups exhibits a negative correlation with the *E*_α_ for CO_2_ generation. An increase in their relative content contributes to a reduction in the *E*_α_ required for CO_2_ formation during the SO process. The main reaction pathways are illustrated in Fig. 13.9
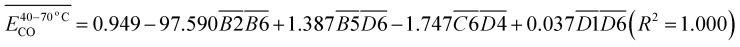


**Fig. 13:**
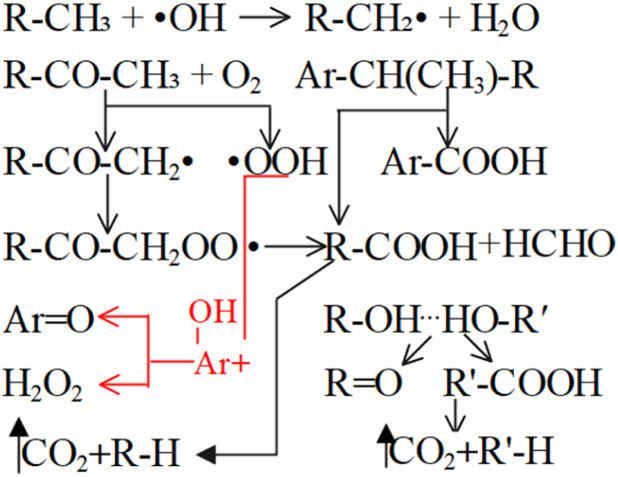
The main reaction pathway of eqn (8).

During the 40–70 °C stage of the SO process of coal, the average *E*_α_ for CO generation 
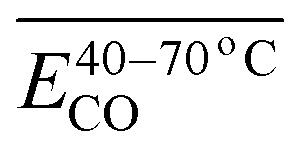
 is predominantly influenced by the functional group combinations 
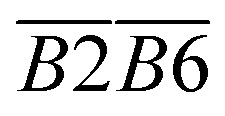
 (–CH_2_/–CH_3_ & car. C=O), 
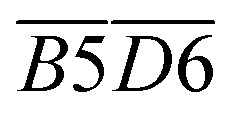
 (quin. C=O & fr. –OH), 
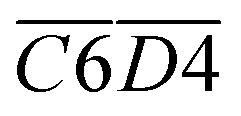
 (*asym*. –CH_3_ & OH⋯OH), and 
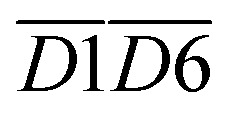
 (aro. C–H & fr. –OH). Among these, the relative content of (–CH_2_/–CH_3_ & car. C=O) exerts the most significant effect on the *E*_α_ for CO generation at this low-temperature stage, exhibiting a negative correlation. A higher product of the two functional groups prior to SO results in lower *E*_α_. This phenomenon primarily arises from the homolytic cleavage of C–H bonds in –CH_2_/–CH_3_, followed by hydrogen transfer, which leads to the formation of unstable free radical intermediates. These intermediates facilitate the cleavage of C=O bonds and the generation of new free radicals. After the break, the C–*R*′ free radicals recombine or combine with *H* , eventually forming CO. This process can significantly reduce the *E*_α_ for CO generation and promote its formation. Similarly, *asym*. –CH_3_ & OH⋯OH also have a negative correlation, but their influence is relatively minor. The primary reason is that *asym*. –CH_3_ (asymmetric methyl) groups are susceptible to free radical formation, whereas OH⋯OH (hydrogen bond networks) may stabilize the molecular structure, thereby increasing the strength of C–H bonds, impeding free radical diffusion, and raising the energy barrier for CO generation. Additionally, quin. C=O & fr. –OH as well as aro. C–H & fr. –OH exhibit a weak positive correlation with the *E*_α_ for CO formation at this temperature stage. An increase in their relative content exerts a minor elevating effect on the *E*_α_ required for CO generation. This phenomenon can be attributed to the fact that quin. C=O (quinone carbonyl) exhibits a strong electron-withdrawing capability, whereas fr. –OH (free hydroxyl) functions as an electron-donating group. These two groups may engage in hydrogen bonding or undergo electron transfer, leading to the detachment of the ˙C=O^+^ fragment and the subsequent formation of CO. The presence of hydrogen bonds increases the energy barrier for CO release, thereby retarding the CO generation process.10
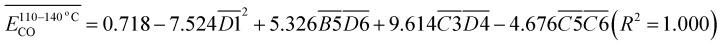


The average *E*_α_ for CO formation in the 110–140 °C stage 
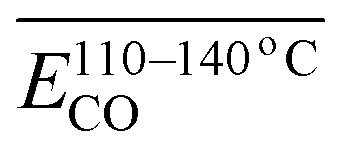
 has a certain correlation with 
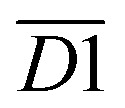
 (the relative content of aro. C–H), 
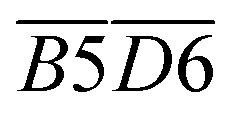
 (the product of the relative contents of quin. C=O and fr. –OH), 
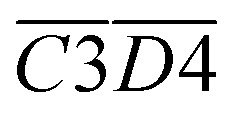
 (the product of the relative contents of *sym*. –CH_3_ and OH⋯OH), and 
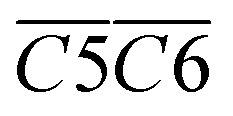
 (the product of the relative contents of *asym*. –CH_2_ and *asym*. –CH_3_). Among them, aro. C–H, *asym*. –CH_2_ and *asym*. –CH_3_ have an enhancing effect on the *E*_α_ for CO formation at this temperature stage. The aromatic C–H bond tends to undergo homolytic cleavage upon heating, resulting in the formation of aromatic radicals (Ar˙), which subsequently react with O_2_ to generate peroxide intermediates that ultimately decompose into CO. However, the fact that this term in eqn (10) is 
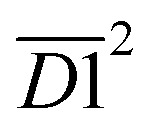
 indicates that when the content of aro. C–H is low, its influence on the *E*_α_ for the formation of CO is relatively weak. With increasing aro. C–H content, the rate of free radical chain reactions enhances, thereby significantly lowering the *E*_α_. Additionally, asymmetric aliphatic chains (*asym*. –CH_2_/CH_3_) are susceptible to β-cleavage, leading to the formation of aldehyde intermediates that further oxidize to CO. This pathway facilitates low-temperature oxidation and contributes to a reduction in *E*_α_. The quinone carbonyl (quin. C=O) is a strong electron-withdrawing group. It forms hydrogen bonds or charge transfer complexes with free hydroxyl groups (fr. –OH), temporarily stabilizing the structure and delaying oxidation. Oxidation requires higher energy to break the hydrogen bonds, thus slightly increasing the *E*_α_. Symmetrical methyl groups (*sym*. –CH_3_) are difficult to form free radicals in the hydrogen bond network (OH⋯OH), and the hydrogen bonds enhance the intramolecular cohesion, significantly increasing the oxidation *E*_α_ and inhibiting the generation of CO.11
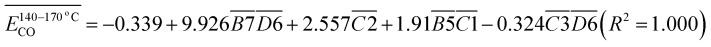


The average *E*_α_ for CO formation during the 140–170 °C stage is 
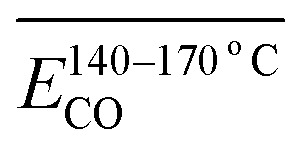
 correlates with the relative contributions of 
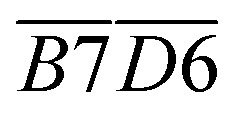
 (C=O & fr. –OH), 
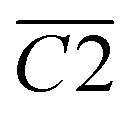
 (*sym*. –CH_2_), 
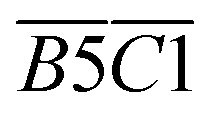
 (quin. C=O & –COOH), and 
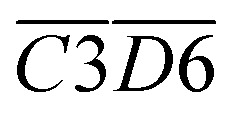
 (*sym*. –CH_3_ & fr. –OH), as described by eqn (11). Among these functional group combinations, C=O & fr. –OH, *sym*. –CH_2_, and quin. C=O & –COOH tend to moderately increase the *E*_α_ required for CO generation at this temperature range. In contrast, only the *sym*. –CH_3_ & fr. –OH interaction demonstrates a slight reducing effect on the *E*_α_ for CO formation. The carbonyl group (C=O) and the free hydroxyl group (fr. –OH) can form intramolecular hydrogen bonds, leading to the formation of a stable six-membered ring transition state. The enhanced stability of this structure necessitates a higher energy input for its cleavage, thereby significantly increasing the *E*_α_. The symmetrical methylene group (*sym*. –CH_2_), characterized by its structural regularity and high C–H bond dissociation energy, exhibits resistance to homolytic cleavage. Consequently, an increase in its content results in a corresponding elevation of the overall *E*_α_. The quinone carbonyl (quin. C=O) and the carboxyl group (–COOH) can establish a conjugated system, forming a stabilized structure represented as O = quin. C=O⋯HOOC–, which requires additional energy to disrupt. In contrast, the interaction between the methyl group (*sym*. –CH_3_) and the free hydroxyl group (fr. –OH) introduces steric hindrance (CH_3_⋯HO–), which weakens the hydrogen bond network (OH⋯OH interactions) and slightly facilitates the generation of free radicals, thereby reducing the *E*_α_.12
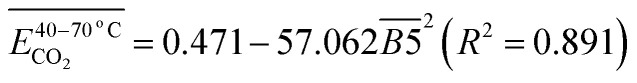


The average *E*_α_ for CO_2_ generation during the 40–70 °C stage is 
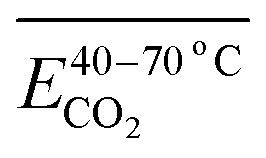
 exhibits a negative correlation with the square of the relative content of quin. C=O (
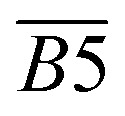
). As the relative content of quin. C=O increases, the *E*_α_ decreases. This phenomenon can be primarily attributed to the fact that the quinone carbonyl group functions as a strong electron-withdrawing moiety. Through conjugation effects, it reduces the electron cloud density along adjacent C–C bonds, thereby weakening their bond strength. This structural modification facilitates the formation of carboxylic acid groups (–COOH), which serve as precursors for CO_2_ generation. Furthermore, the quinone carbonyl group participates in synergistic oxidation reactions with neighboring aliphatic chains, ultimately leading to the production of CO_2_. Throughout this progressive oxidation process, the reaction energy barriers are systematically lowered, resulting in a continuous decrease in the *E*_α_ required for CO_2_ formation within this temperature range. However, in eqn (12), the quin. C=O term is represented by the square of its relative content 
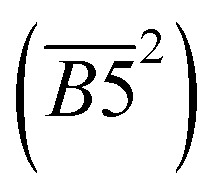
, indicating that at low relative contents of quin. C=O, its impact on the *E*_α_ for CO_2_ generation during this temperature stage is negligible. A substantial reduction in the *E*_α_ for CO_2_ formation is only observed when the relative content of quin. C=O reaches a relatively high level.13
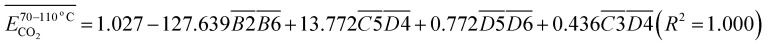

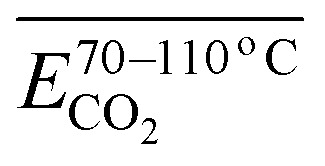
 is the average *E*_α_ of CO_2_ generation in the 70–110 °C temperature range, eqn (13) reveals that only the functional group combination 
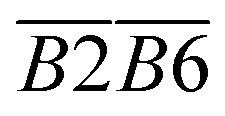
 (–CH_2_–CH_3_ & car. C=O) exhibits a significant negative correlation, with a relatively high regression coefficient of 127.639. This suggests that even a minor increase in the relative content of (–CH_2_–CH_3_ & car. C=O) can lead to a notable reduction in the *E*_α_ required for CO_2_ formation. The underlying mechanism is likely attributed to the synergistic oxidation process between the aliphatic chain and the conjugated carbonyl group. Specifically, the aliphatic chain serves as a hydrogen donor, while the carbonyl group activates the adjacent C–H bond, facilitating the formation of peroxides and subsequent generation of R–COOH species. As a result, the energy barrier associated with this reaction pathway is relatively low, indicating that this process constitutes a primary route for CO_2_ production. (R–CH_2_–CH_3_ + car. C=O + O_2_ → R–CH_2_–C(OOH)–CH_3_ → R–COOH + CH_3_COOH → CO_2_)Other indicators, such as 
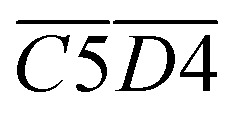
 (*asym*. –CH_2_ & OH⋯OH), 
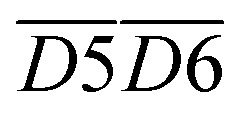
 (OH⋯π & fr. –OH), and 
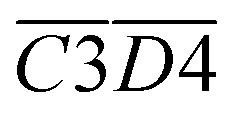
 (*sym*. –CH_3_ & OH⋯OH), exhibit a positive correlation with 
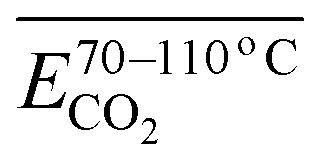
*E*_α_. That is, an increase in their relative contents may lead to a corresponding increase in *E*_α_. For instance, the 
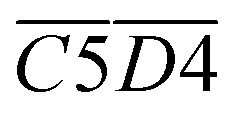
 interaction involving *asym*. –CH_2_ may elevate *E*_α_ primarily due to the inhibitory effect of the hydrogen bond network on oxidation. The intermolecular hydrogen bonding between hydroxyl groups (OH⋯OH) contributes to molecular structural stability. The *asym*. –CH_2_ bonds themselves require higher energy for cleavage, and any interaction involving these bonds necessitates prior disruption of hydrogen bonds. Therefore, their coexistence and simultaneous increase in relative content result in a higher *E*_α_ required for CO_2_ generation through oxidation. Additionally, OH⋯π interactions compete with fr. –OH groups, where the OH⋯π hydrogen bonds disperse radical attack sites, requiring the prior dissociation of π bonds. Similarly, the oxidation of *sym*. –CH_3_ groups must overcome steric hindrance. Collectively, these processes demand relatively high *E*_α_.14
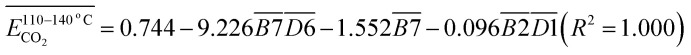

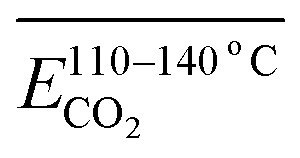
 denotes the average activation energy for CO_2_ generation during the temperature stage of 110–140 °C (eqn (14)). In this process, the product of the relative contents of 
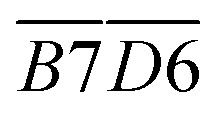
 (–C=O & fr. –OH) serves to supply protons from free hydroxyl groups (fr. –OH), while the carbonyl group (–C=O) activates the β position C–C bond, synergistically promoting the decarboxylation reaction through the following pathway: R–CO–CH_2_– + fr. –OH → R–CO–CH– + H_2_O → R–COOH → CO_2_; 
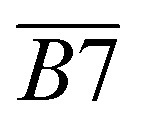
 represents the relative content of isolated carbonyl groups (C=O), which undergo direct cleavage, as well as the low-temperature decarboxylation of quinone-like structures, resulting in a reaction rate enhancement of up to 10^3^ fold. 
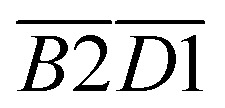
 represents the product of the relative contents of –CH_2_\–CH_3_ and aro. C–H, indicating the cooperative effect between aliphatic chains and aromatic rings in facilitating oxidation reactions. Ar–H + –CH_2_–CH_3_ → Ar– + –CH_2_–CH_2_– → Peroxide → Aldehyde → CO_2_; The relative content of the relevant functional groups involved in the above process has a negative correlation with the *E*_α_. As the relative content increases, the *E*_α_ decreases.15
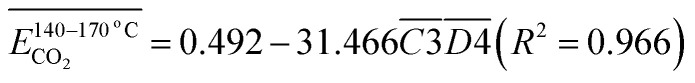


The average *E*_α_ of CO_2_ generation in the 140–170 °C stage is denoted as 
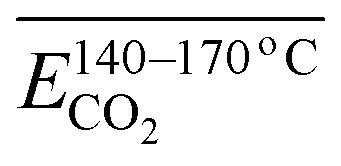
. Eqn (15) shows that 
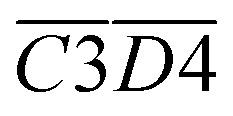
 (*sym*. –CH_3_ & OH⋯OH) has a negative correlation with 
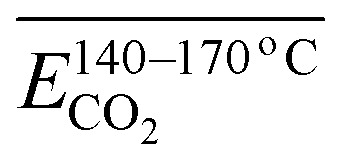
 indicating that an increase in the relative content of *sym*. –CH_3_ & OH⋯OH can reduce the *E*_α_ of CO_2_ generation at this temperature stage. The primary mechanism underlying this phenomenon involves the activation of methyl groups *via* proton transfer within the hydrogen bond network, leading to the formation of the free radical CH_2_–R. The OH⋯OH hydrogen bonding structure contributes to the stabilization of the transition state and enhances the efficiency of proton transfer, thereby significantly reducing the *E*_α_ required for methyl dehydrogenation (C–H bond cleavage). The spatial symmetry of the *sym*. –CH_3_ group facilitates its interaction with the hydrogen bond network, further accelerating the dehydrogenation process and promoting free radical generation. Subsequently, peroxide chain reactions and deep oxidation pathways proceed through low-energy-barrier mechanisms, enabling the efficient conversion of carbon chains into CO_2_. These sequential and synergistic processes collectively contribute to a substantial reduction in the *E*_α_ for CO_2_ generation.

## 4 Conclusions

(1) During secondary oxidation (SO) over 40–170 °C, the apparent activation energy (*E*_α_) for CO evolution is predominantly governed by two opposing functional group interactions: (i) the –CH_2_/–CH_3_ & carbonyl (C=O) pair, which significantly lowers *E*_a_*via* formation of a six-membered cyclic transition state-facilitated by facile oxidation of the α-carbon adjacent to the carbonyl group; and (ii) the –CH_2_/–CH_3_ & asymmetric –CH_3_ pair, which elevates *E*_α_ due to steric congestion that impedes H-abstraction from the hindered methyl site. In contrast, *E*_α_ for CO_2_ evolution across the same temperature interval is jointly modulated by –CH_2_/–CH_3_ & C=O, asymmetric –CH_3_, and intermolecular hydrogen-bonded motifs (OH⋯OH and OH⋯π). All exhibit strong negative correlations with CO_2_*E*_α_, with the –CH_2_/–CH_3_ & C=O interaction exerting the largest suppressive effect-consistent with its dual role in promoting both decarbonylation and carboxylate decomposition pathways.

(2) Temperature-resolved analysis reveals distinct structure-reactivity regimes: (i) in the low-temperature SO stage (40–70 °C), the average *E*_α_ for CO generation is most sensitively suppressed by elevated –CH_2_/–CH_3_ & C=O content, while quinonoid C=O & free-OH, asymmetric –CH_3_ & OH⋯OH, and aromatic C–H & free –OH combinations also contribute significantly. (ii) In the mid-temperature range (110–140 °C), *E*_α_ exhibits strong negative correlations with aromatic C–H, asymmetric –CH_2_, and asymmetric –CH_3_-indicating enhanced radical initiation from these sites-yet shows positive correlations with quinonoid C=O & free –OH and symmetric –CH_3_ & OH⋯OH, suggesting competitive stabilization or kinetic inhibition under these conditions. (iii) In the high-temperature window (140–170 °C), *E*_α_ is strongly and positively correlated with C=O & free –OH, symmetric –CH_2_, and quinonoid C=O & –COOH, but weakly and negatively correlated with symmetric –CH_3_ & free–OH–highlighting a shift toward acid-catalyzed and symmetry-facilitated dehydrogenation mechanisms.

(3) For CO_2_ evolution, functional group effects are markedly temperature-dependent: (i) at 40–70 °C, *E*_α_ scales inversely with the square of quinonoid C=O concentration-indicating second-order kinetic dependence and pointing to quinone-mediated electron-transfer catalysis in early-stage carboxylation. (ii) Between 70–110 °C, *E*_α_ is strongly suppressed by –CH_2_/–CH_3_ and carbonyl (C=O) groups, while exhibiting weak positive correlations with asymmetric –CH_2_, OH⋯OH, OH⋯π & free –OH, and symmetric –CH_3_ & OH⋯OH–suggesting competing roles of H-bonding networks in either stabilizing intermediates or retarding proton-coupled electron transfer. (iii) In the 110–140 °C regime, the dominant factor is the C=O & free –OH interaction: free-OH acts as a Brønsted proton donor, while adjacent C=O polarizes and activates the β–C–C bond-synergistically lowering the decarboxylation barrier through concerted proton transfer and bond cleavage. (iv) At 140–170 °C, *E*_α_ is primarily dictated by symmetric –CH_3_ & OH⋯OH interactions. The extended hydrogen-bond network enables efficient proton relay, stabilizing dehydrogenation transition states and enhancing methyl radical formation. Critically, the spatial symmetry of –CH_3_ improves geometric compatibility with the H-bond lattice, accelerating initial C–H homolysis and enabling rapid propagation of peroxide chain reactions-thereby facilitating deep oxidative cleavage of aliphatic chains into CO_2_ at substantially reduced energetic cost.

## Conflicts of interest

The authors declare no competing financial interest.

## Data Availability

All relevant data utilized in this article are encompassed within the figures and data tables presented in the manuscript.
